# High-Throughput Epitope Binning Assays on Label-Free Array-Based Biosensors Can Yield Exquisite Epitope Discrimination That Facilitates the Selection of Monoclonal Antibodies with Functional Activity

**DOI:** 10.1371/journal.pone.0092451

**Published:** 2014-03-20

**Authors:** Yasmina Noubia Abdiche, Adam Miles, Josh Eckman, Davide Foletti, Thomas J. Van Blarcom, Yik Andy Yeung, Jaume Pons, Arvind Rajpal

**Affiliations:** 1 Rinat-Pfizer Inc, South San Francisco, California, United States of America; 2 Wasatch Microfluidics, Salt Lake City, Utah, United States of America; Technical University of Braunschweig, Germany

## Abstract

Here, we demonstrate how array-based label-free biosensors can be applied to the multiplexed interaction analysis of large panels of analyte/ligand pairs, such as the epitope binning of monoclonal antibodies (mAbs). In this application, the larger the number of mAbs that are analyzed for cross-blocking in a pairwise and combinatorial manner against their specific antigen, the higher the probability of discriminating their epitopes. Since cross-blocking of two mAbs is necessary but not sufficient for them to bind an identical epitope, high-resolution epitope binning analysis determined by high-throughput experiments can enable the identification of mAbs with similar but unique epitopes. We demonstrate that a mAb's epitope and functional activity are correlated, thereby strengthening the relevance of epitope binning data to the discovery of therapeutic mAbs. We evaluated two state-of-the-art label-free biosensors that enable the parallel analysis of 96 unique analyte/ligand interactions and nearly ten thousand total interactions per unattended run. The IBIS-MX96 is a microarray-based surface plasmon resonance imager (SPRi) integrated with continuous flow microspotting technology whereas the Octet-HTX is equipped with disposable fiber optic sensors that use biolayer interferometry (BLI) detection. We compared their throughput, versatility, ease of sample preparation, and sample consumption in the context of epitope binning assays. We conclude that the main advantages of the SPRi technology are its exceptionally low sample consumption, facile sample preparation, and unparalleled unattended throughput. In contrast, the BLI technology is highly flexible because it allows for the simultaneous interaction analysis of 96 independent analyte/ligand pairs, *ad hoc* sensor replacement and on-line reloading of an analyte- or ligand-array. Thus, the complementary use of these two platforms can expedite applications that are relevant to the discovery of therapeutic mAbs, depending upon the sample availability, and the number and diversity of the interactions being studied.

## Introduction

In the quest for therapeutic monoclonal antibodies (mAbs), the selection of appropriate affinity, specificity and biophysical properties is essential. Methodologies that allow an optimal candidate to be selected from a large number of leads can make the difference between a successful program and a clinical failure, even when the target has been properly chosen. A mAb's epitope correlates with its functional activity [1], [2], but the *in silico* prediction of B-cell epitopes is not yet possible [3], so epitope selection remains an empirical process. Early-stage drug discovery efforts often generate large panels of mAbs per target via complementary approaches such as traditional hybridoma and modern phage-display methods, so it is helpful to organize mAbs into epitope families or “bins”. MAbs that target similar epitopes often share a similar function, so identifying an epitope bin with functional activity provides several potential leads to choose from. Conversely, if mAbs from multiple epitope bins exhibit functional activity, this may imply different mechanisms of action, which can be advantageous when pursuing an oligoclonal therapy to treat some cancers or infectious diseases where simultaneously targeting more than one biological pathway may be needed [4]–[8]. With the high cost of developing a therapeutic mAb, the ability to identify a few high quality leads with relevant epitopes early in the discovery process cannot be overstated.

**Figure 1:**
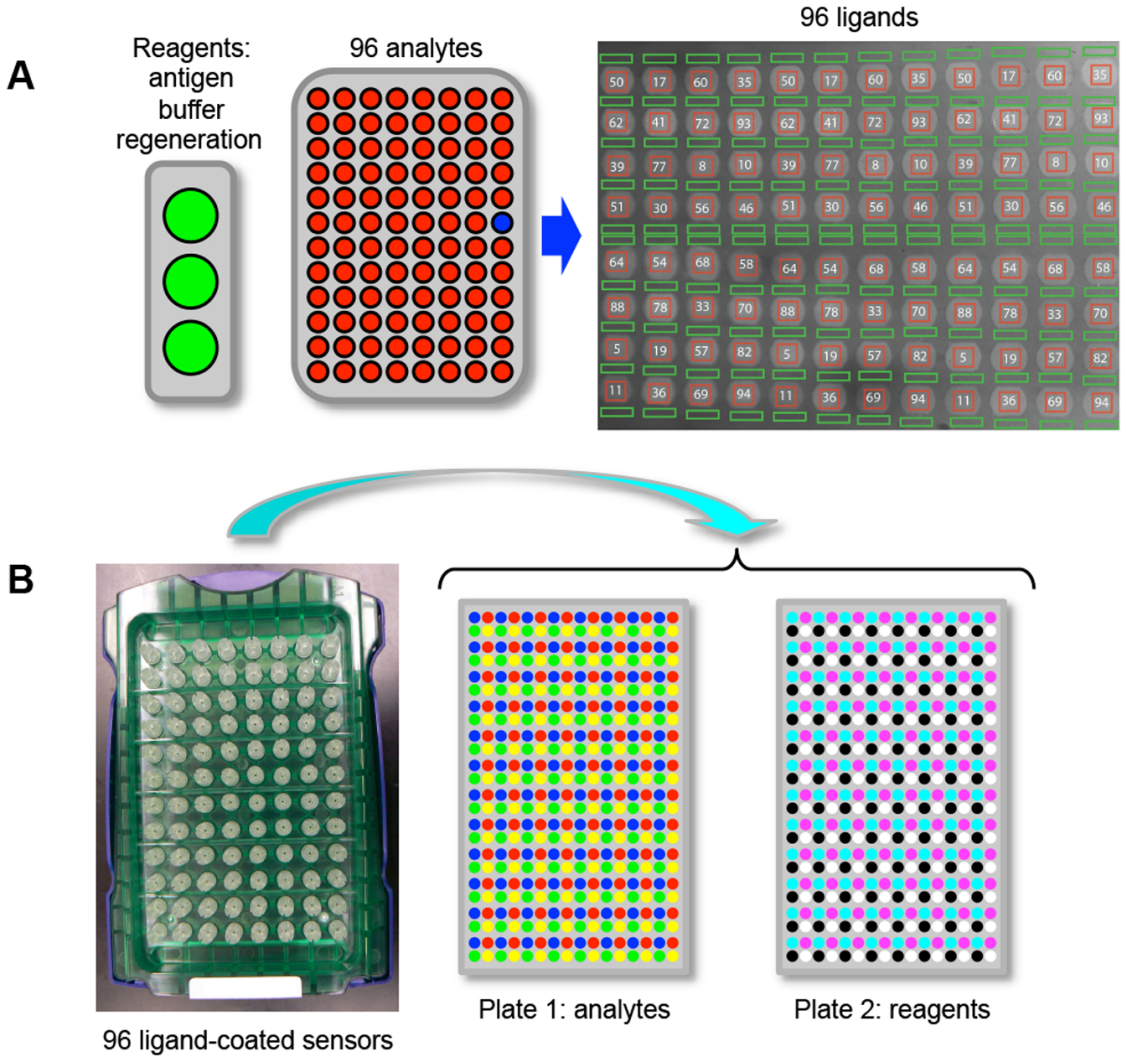
Assay set-up and autosampler capacity of two array-based biosensors. (A) A CFM is used to print a microarray of 96 mAbs onto a sensor chip, which is then loaded into a SPRi reader. In the image of the 96-ligand array shown here, the red squares define the reaction spots (named by mAb) and the green rectangles define the interstitial reference spots (two per adjacent reaction spot). The SPRi is equipped with an autosampler that injects an analyte and cycles it back-and-forth across the flow cell. Up to 96 analytes are accommodated in a 96-well microplate and common reagents (i.e., antigen, buffer, and regeneration) are accommodated in 11-ml vials. (B) In the BLI system, 96 ligand-coated fiber optic sensors are dipped in parallel into a 96-analyte array. Analytes and common reagents are distributed across two 384-well microplates. The sample layout depicts that used for a typical classical sandwich epitope binning assay (see **Table 1**).

While determining the crystal structure of an antigen/mAb complex is the recognized “gold standard” method for defining an epitope with precision at the molecular level, it is low-throughput, labor-intensive, and requires large amounts of highly pure reagents. Therefore, it is not amenable to early-stage research where efforts focus on selecting leads for further characterization. Epitope binning assays on label-free biosensors are an attractive approach for discriminating mAbs in a test panel based upon their binding to a specific antigen because they can be performed at relatively low cost and high throughput without the need for specialized reagents; only the mAbs and the antigen of interest are required. Various multiplexed array-based platforms are currently available from the leading vendors of commercial biosensors (e.g., Biacore from GE Healthcare, ProteOn from BioRad, and Octet from FortéBio, a division of Pall Life Sciences). Until recently, they have been limited to processing 36 or fewer interactions simultaneously and by the number and diversity of analyte/ligand interaction pairs that could be explored per unattended assay as a consequence of autosampler capacity and the assay configurations that are amenable on each platform [9]. To address the ever-increasing demands on the drug discovery industry for assays that are both higher throughput and more informative, we evaluated two state-of-the-art biosensors that each enable the simultaneous analysis of 96 analyte/ligand interactions. The first platform uses continuous flow microspotting (CFM) technology [10] to immobilize 96 ligands on a sensor chip, which is then read via surface plasmon resonance imaging (SPRi) within a single flow cell of the IBIS-MX96 instrument. Analytes are accommodated in a 96-well microplate and microfluidics are used to inject them one after another over the 96-ligand array, thereby performing an interaction analysis on 9216 unique analyte/ligand pairs per experiment (**Figure 1A**). The second platform is the Octet-HTX, which is a higher-throughput version of the well-established biolayer interferometry (BLI)-based Octet-Red384 platform. In the Octet-HTX, 96 ligand-coated sensor tips dip into a 96-analyte array thereby addressing 96 independent analyte/ligand interactions in parallel. Since the BLI system does not employ microfluidic sample handling, all samples including analytes and common reagents (antigen, buffer and regeneration solutions) are accommodated within two 384-well microplates (**Figure 1B**). While neither system provides high-resolution kinetic data compared with established Biacore technology, the throughput of these instruments is orders of magnitude higher, thereby providing excellent tools for functional antibody characterization.

SPR and BLI are complementary optical phenomena. SPR is dependent on the refractive index at the sensor surface, which is directly proportional to the mass of analyte bound. Therefore, an analyte's maximum theoretical binding response can be calculated if one knows the molecular weights of the analyte and ligand, their binding stoichiometry, and the level of immobilized ligand. In contrast, BLI is dependent on the interference of white light from two surfaces within a single biosensor that consists of a protein layer (the ligand) and an internal reference layer. The binding of analyte to the ligand causes a change in the optical thickness at the biosensor surface, which shifts the interference pattern and generates a response that is dependent on both the number of analyte molecules bound and their orientation. Therefore, BLI does not yield data that is linearly dependent on the mass ratio of a given analyte/ligand pair, so large analytes may give smaller signals than one would expect by SPR, and vice versa.

An epitope binning experiment involves competing mAbs against one another in a pairwise and combinatorial fashion for binding to a specific antigen. An epitope bin is a relative concept based upon the epitopes represented within the panel of mAbs being tested. Thus, two mAbs belong to the same bin if they share the same blocking profile when tested against all other mAbs in the test panel. Since the number of interactions required for a comprehensive epitope binning analysis scales geometrically with the number of mAbs in the test panel (i.e., binning ten mAbs requires 100 interactions; and binning 100 mAbs requires 10,000 interactions) an epitope binning assay rapidly escalates into a large experiment. High-throughput epitope binning assays on large panels of mAbs have the potential to yield high-resolution binning information because the discriminating power of the assay increases with the epitopic diversity within the test panel.

There is no standard nomenclature to describe epitope binning assay formats. Among the numerous terms used in the literature are, epitope mapping [11], sandwiched immunoassay [12], IgG blocking [13], IgG competition [14], solution competition and surface competition [15]. Herein, we adhere to our previous terminology; classical sandwich, premix, and in tandem [16], [17]. Briefly, in a classical sandwich assay, a mAb analyte is tested for binding to antigen that is first captured via an immobilized mAb. In a premix assay, the antigen is saturated with a mAb analyte in solution and this preformed antigen/mAb complex is tested for binding to an immobilized mAb; its binding response is compared with that for antigen alone. An in tandem assay involves binding two mAbs, one after another, to an immobilized antigen. Thus, a mAb analyte is competed against an immobilized mAb in both the classical sandwich and premix assay formats, whereas no mAbs are immobilized in the in tandem approach. Within a given assay format, every pairwise permutation of mAbs is tested to increase the confidence of the binning result because, for a given pair of mAbs, switching the order in which they bind their antigen may give a clearer outcome [17], [18].

Here, we demonstrate the fine epitope discrimination that can be determined from epitope binning assays on large panels of mAbs. We then use this information to show that epitope bins correlate with functional activity, thereby strengthening the biological relevance and highlighting the predictive power of epitope binning assays that are both high throughput and high resolution in guiding the selection and development of therapeutic mAbs.

## Materials and Methods

### General reagents

Purified recombinant human progranulin (rhPGRN, catalog number 2420-PG) and biotinylated anti-His mAb (catalog number BAM050) were obtained from R&D Systems (Minneapolis, MN). Purified recombinant *Staphylococcus aureus* iron-regulated surface determinant protein B (rIsdB) with a C-terminal 8-His-tag and an N-terminal Flag-tag and Avi-tag and a predicted molecular mass of approximately 68 kDa was prepared in-house. Anti-hPGRN and anti-IsdB mAbs were generated and purified in-house. Hemoglobin (Hb) was purified in-house from fresh red blood cells obtained from Bioreclamation LLC (Westbury, NY). Briefly, Hb from cell lysates was purified using Q-sepharose XL resin (GE Healthcare), followed by Superdex 200 Prep Grade resin (GE healthcare) for size exclusion chromatography [19]. Anti-Flag mAb (catalog number F3165) and Flag peptide (catalog number F3290) were purchased from Sigma Inc., St Louis, MO. Anti-Flag mAb was biotinylated in-house using a five-fold molar excess of EZ link NHS-LC-LC-biotin (catalog number 21343, Pierce Biotechnology, Rockford, IL). IgG elution buffer pH 2.8 and amine-coupling activation reagents were also purchased from Pierce; activation reagents were stored at −20°C as single-use aliquots at stock concentrations of 0.4 M 1-ethyl-3-[3-dimethylaminopropyl]-carbodiimide hydrochloride (EDC) and 0.1 M *N*-hydroxysulfosuccinimide (sulfo-NHS) in water. Amine-coupling buffers (0.1 M MES pH 4.5 or pH 5.0) were prepared using 2-(N-morpholino)ethanesulfonic acid sodium salt (MES, catalog number M3058, Sigma). Amine-coupling blocking reagent (1 M ethanolamine HCl pH 8.5) was purchased from GE Healthcare. All experiments were conducted at 25°C in PBS buffer supplemented with 0.01% Tween-20 (SPRi experiments) or 0.05% Tween-20 + 1 g/l BSA (BLI experiments). Coupled mAbs were regenerated using 75 mM phosphoric acid, unless stated otherwise.

### BLI assays

Octet systems (HTX, Red384, and QK384) equipped with amine-reactive, streptavidin, and anti-species sensors were purchased from Pall Life Sciences (Menlo Park, CA). Epitope binning experiments were performed in 96-channel mode (HTX system) or 16-channel mode (Red384 and QK384 systems). MAbs were coupled onto amine-reactive sensors on-line using a standard protocol in MES coupling buffer (either pH 4.5 or pH 5.0, depending upon the experiment). Briefly, sensors were soaked in coupling buffer (30 min), activated in a freshly prepared mixture of EDC and sulfo-NHS (each diluted tenfold in MES buffer from their stock concentrations), coupled with 30 μg/ml mAb, and excess reactive esters were blocked with ethanolamine. Activation, coupling, and blocking steps were allowed 15 min each.

To perform a classical sandwich epitope binning assay, each binding cycle consisted of four steps: 1) a baseline was established in running buffer for 3 min, 2) antigen (5 nM rhPGRN or 10 nM rIsdB) was captured for 10–15 min, 3) mAb analyte (5 μg/ml) was bound for 5–15 min, and 4) the surfaces were regenerated for 30–45 sec. The timing of the binding steps varied, depending upon the experiment. For the anti-IsdB mAbs, the regeneration solutions were optimized per mAb from the following panel (15 mM, 30 mM or 75 mM phosphoric acid; or 6 mM NaOH + 1 M NaCl).

Performing a premix epitope binning assay on sensors coupled with anti-hPGRN mAbs involved a three-step binding cycle; 1) a baseline was established for 3 min, 2) mixtures of 10 nM rhPGRN with or without 200 nM binding sites of each anti-hPGRN mAb were bound for 15 min, and 3) surfaces were regenerated for 30 sec.

To generate surfaces for in tandem epitope binning assay on rIsdB, streptavidin sensors were coated with 2 μg/ml biotinylated capture reagent (anti-His mAb or anti-Flag mAb) for 30 min. The in tandem style assay comprised a five-step binding cycle; 1) a buffer baseline was established for 3 min, 2) 5 μg/ml rIsdB was captured for 5–15 min, 3) 20 μg/ml mAb array was loaded to saturate the immobilized antigen for 5–15 min, 4) 10–20 μg/ml of the test mAb was bound for 5–15 min, and 5) the capture surfaces were regenerated for 30 sec. Anti-His surfaces were regenerated with 75 mM phosphoric acid and anti-Flag surfaces were regenerated using 2∶1 v/v Pierce IgG elution buffer/4 M NaCl supplemented with 0.1 g/l Flag peptide. In tandem assays were conducted on all three Octet systems (HTX, Red384, and QK384), depending on the experiment.

The anti-IsdB mAbs were tested for their ability to block the rIsdb/Hb interaction using a classical sandwich assay format as follows; 15 μg/ml mAbs were captured via anti-species sensors, 32 nM rIsdB was bound, and then 1 μM Hb was tested, allowing 10 min per step. The sensors were regenerated for 30 sec after each binding cycle. An isotype-matched negative control mAb was used to monitor any non-specific cross-reaction of rIsdB or Hb.

### SPRi and CFM method

A CFM 2 (Wasatch Microfluidics) was used to create a microarray of 96 mAbs. It draws forty-eight 70-μl plugs of sample from a 96-well microplate into a fluidic manifold which focuses the solutions into a 4×12 array of 48 micro flow cells on the surface of the SPR substrate (a G-COOH coated prism from Ssens bv, NL) and cycles the solutions back and forth at 60 μl/min. A 96-well microplate was prepared with 100 μl of each mAb at 30 μg/ml in MES coupling buffer pH 4.5 and loaded into bay 2 of the CFM. A second plate of freshly mixed activating reagents (150 μl 0.4 M EDC and 150 μl 0.1 M sulfo-NHS in a total of 5 ml of MES coupling buffer pH 4.5) was loaded into bay 1. The CFM was then primed with system buffer (PBS + 0.01% T20). The anti-hPGRN mAb plate contained six replicate sets of sixteen mAbs, and the anti-IsdB mAb plates contained either 32 mAbs arrayed in triplicate or 24 mAbs arrayed in quadruplicate. Once docked, the activating reagents were cycled over the surface for 7 min and followed immediately by the first set of 48 mAbs (top half of the mAb plate) and cycled for 15 min. Without undocking, the spots were rinsed with the system buffer. Since the CFM prints 48 solutions at a time, it needs to address the surface twice to create the full 8×12 array of 96 mAbs. After the first print, the CFM was paused to load fresh activation reagents, and the same cycle of 7-min activation and 15-min coupling was repeated for the second half of the mAb plate.

The printed prism was then loaded into the SPRi reader (MX96, IBIS Technologies bv), which uses a single flow cell and autosampler configured to address the array with back-and-forth cycled injections of 80 μl per analyte. Once loaded, 1 M ethanolamine was injected across the chip for 15 min to quench the excess reactive esters. The chip was then washed with system buffer and the chip image was used to define the reaction spots (i.e., the 96-ligand array) and the interstitial reference spots (two local reference spots per reaction spot). For the premix style binning assay, a standard injection cycle of analyte and regeneration was used. For classical binning, a co-injection was used, where both antigen and mAb analyte were transported to the flow cell in parallel lines, and injected immediately after one another before continuing with regeneration. For classical binning experiments, antigen (16 nM rhPGRN or 35 nM rIsdB) was injected for 3 min, followed by 20 μg/ml mAb for a further 3 min, and then the surfaces were regenerated. For the rhPGRN premix binning experiment, samples were prepared by incubating 8 nM rhPGRN with each mAb in a 10–20 fold molar excess and then these equilibrated mixtures were injected for 10 min followed by a regeneration step. All SPRi experiments were conducted in a 96×96 analyte-on-ligand format.

### Biosensor data analysis

Octet data were processed in ForteBio's data acquisition software v. 8.0.0.99 by aligning the sensorgrams to zero on the Y-axis prior to the binding step of interest and analyzed by visual inspection to create a heat map. In the heat maps, the rows represent the ligands and the columns represent the analytes, in the same order. A cell represents an analyte/ligand pair and is color-coded by its blocking status, where red - blocked, green - not blocked, and yellow - intermediate or ambiguous response. Self-blocks are outlined with a bold box. A conflicting blocking result for a given mAb pair (i.e., blocks in only one direction of the heat map) is indicated with a dotted border around those cells. Grey rows represent inactive ligands and grey columns indicate analytes that were not run due to insufficient sample. SPRi data were processed in SPRint software v. 6.15.2.1 (calibrated, locally referenced, and aligned to zero on the Y-axis prior to the binding step of interest) and analyzed in Wasatch Microfluidics' binning software for heat map generation, sorting and node plotting. Hierarchical clustering was used to group like-behaved mAbs together in the heat map. Heat maps and node plots are alternate ways of visualizing epitope bins and their inter-bin relationships. In interpreting a node plot, it is assumed that all mAbs in the panel have been tested for pairwise competition against one another so that a chord connects two mAbs that showed a blocking relationship, and no chord represents a non-blocking relationship. MAbs that belong to the same bin are inscribed by the envelopes (the colors of the envelopes are auto-generated in the binning software and carry no meaning). The node plots for our rIsdB study are color-coded by each mAb's Hb-blocking status, as determined by BLI, where red - blocker, green - non-blocker, and yellow - partial blocker.

### Cell-based assays

The binding of human Hb to endogenously-expressed IsdB on *S. aureus* cells was used to assess the blocking effect of a panel of anti-IsdB mAbs as described by Pishchany et al. [20] with the following modifications. The *S. aureus* ΔSpA strain was used [21]. Anti-IsdB mAbs at a concentration of 600 nM were incubated with *S. aureus* cells for 10 min at room temperature before purified human Hb was added to give a final Hb concentration of 150 nM. Detection of Hb was performed by standard Western blotting with a biotinylated primary antibody (sheep polyclonal anti-Hb, biotinylated, Abcam ab95152, used at 2 μg/ml) followed by a streptavidin-conjugated secondary reagent (Streptavidin-IRDye 800 CW, 1 g/l; Odyssey 926-32230, 1∶4000 dilution). A Lycor Odissey system was used to image the blot and to quantify the band intensities, data were expressed as % of maximum Hb binding in absence of mAbs.

## Results

### Both the SPRi and BLI platforms are amenable to classical sandwich and premix epitope binning assay formats and the results are concordant and independent of the biosensor platform and the assay format employed

**Figure 2:**
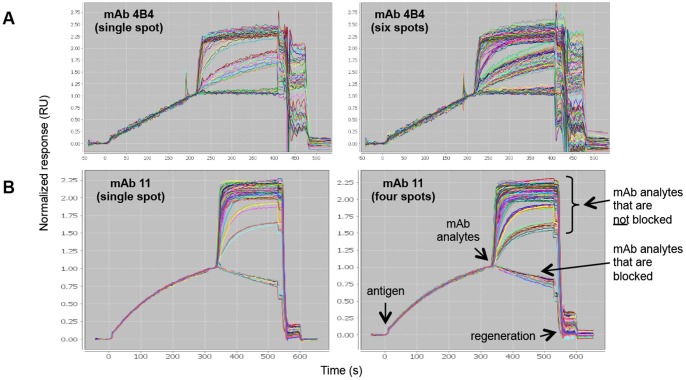
SPRi analysis of epitope binning assays on independent arrays using a classical sandwich assay format. The model antigens used were (A) rhPGRN and (B) rIsdB. Each overlay plot shows the sensorgrams for 100 binning cycles (i.e., 96 mAb analytes and buffer blank analytes) on a single spot (left) or replicate spots (right) of a coupled mAb, as indicated. Responses are normalized to 1 at the end of the antigen capture step to facilitate the comparison of different capacity spots.

Using the SPRi technology, we conducted a series of epitope binning experiments in a classical sandwich assay format on two panels of mAbs that target unrelated monomeric antigens, namely recombinant human progranulin (rhPGRN) and recombinant *Staphylococcus aureus* iron-regulated surface determinant protein B (rIsdB). Having fewer than 96 mAbs per panel enabled us to test both the spot-to-spot and injection-to-injection reproducibility of our experiments and to explore various densities of a given mAb ligand within an array. **Figure 2** shows data obtained by SPRi for two independent experiments on rhPGRN (panel A) and rIsdB (panel B), in which 96 mAb analytes were injected in succession over a 96-ligand mAb array. For mAbs that were facile to regenerate, we obtained data that showed highly reproducible antigen capture on individual spots (**Figure 2** **- left panels**) and across multiple spots that were coupled with the same mAb at different densities (**Figure 2** **- right panels**). The runtime of each experiment that addressed an entire 96×96 analyte-on-ligand interaction matrix was about 30 hours, using standard injection times (**Table 1**).

**Table 1:** Comparison of SPRi and BLI technologies with respect to throughput and sample consumption in performing a comprehensive classical sandwich epitope binning assay on 96

Parameter	SPRi	BLI*
**Time to couple 96 ligands**	1 h (two consecutive prints of a 48-channel CFM followed by blocking of the 96-ligand array in the SPRi)	40 min (one cycle)
**Vol. per ligand (96 total)**	100 μl	100 μl**
**Vol. per analyte (96 total)**	120 μl	10 ml (96× 100-μl aliquots per mAb analyte within a 384-well plate)
**Plates required to accommodate 96 mAb analytes**	One 96-well plate	24× 384-well plates
**Time to prepare 96 analytes**	30 min (dispense 100 μl per mAb into a 96-well plate)	Several hours to dispense 96 mAbs into 24 plates, each plate accommodating four mAbs, and each mAb dispensed into 96× 100 μl-aliquots
**Unattended throughput**	9216 interactions (96 analytes × 96 ligands)	384 interactions (four analytes × 96 ligands) if one plate is used for analytes and the second plate is used for reagents.
**Unattended runtime**	30 h using standard injection times	1 h using four 15-min cycles
**No. experiments needed**	One	24
**Total runtime**	30 h	Several days involving 24 manual plate switches
**Reagents (antigen, buffer, and regeneration)**	10 ml each accommodated in 11-ml vials	10 ml each, but must be distributed as 96× 100-μl aliquots into a 384-well plate

* Robotic integration with the BLI platform results in a higher unattended throughput, longer unattended runtime, and significantly shorter total runtime by automating multi-plate analyses. Less time is needed for sample preparation if a robot is used for the microfluidic dispensing.

**Sample volumes can be reduced by 50% if tilted-bottom 384-well microplates that hold a minimum of 40 μl/well are used instead of the standard flat-bottom 384-well microplates.

**Figure 3:**
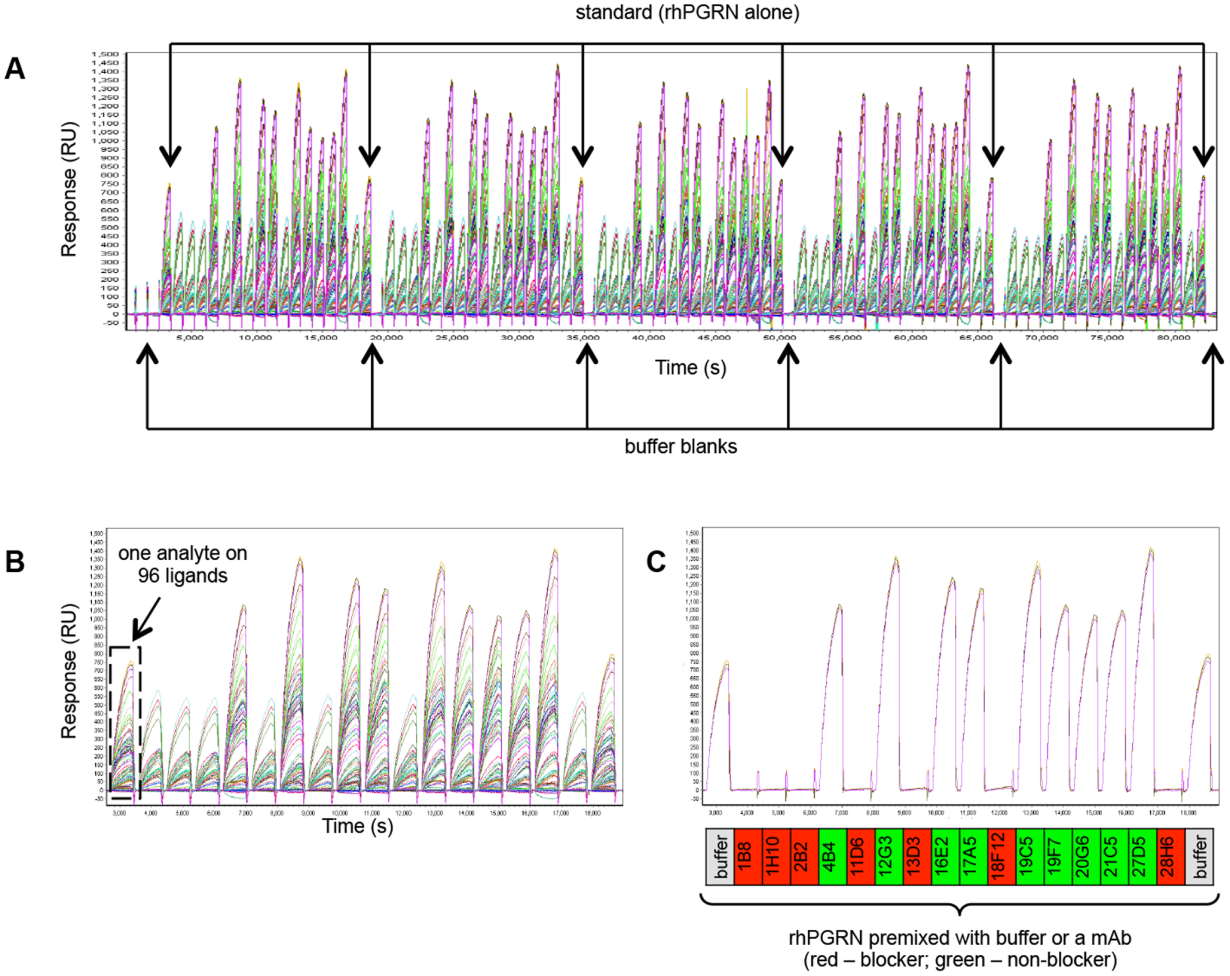
SPRi analysis of sixteen anti-hPGRN mAbs using a premix epitope binning assay format. (A) Serial view of the sensorgrams obtained for 94 analytes (i.e., five replicate sets of rhPGRN/mAb premixed samples, interspersed with replicates of rhPGRN alone and buffer blanks), (B) zoomed in view of the first set of analytes, and (C) same view as panel B, but showing an overlay plot for three spots coupled with mAb 1B8 at similar capacities (the other three spots coupled with mAb 1B8 had different capacities because those surfaces were prepared in a subsequent print; they are excluded for clarity). Response data is aligned to zero at the start of each analyte injection and the regeneration steps have been excluded from view.

We further studied the anti-hPGRN mAbs by SPRi in a premix assay format. **Figure 3A** shows a concatenated view of the sensorgrams obtained for 96 analytes consisting of five replicate sets of hPGRN premixed separately with each of the sixteen mAbs, interspersed with samples of rhPGRN alone and buffer blanks injected over a 96-ligand array comprised of sixteen mAbs coupled onto six spots each. **Figure 3B** and **Figure 3C** show an expanded view of the data obtained for the first set of analytes over the entire 96-ligand array (panel B) or over three spots coupled in parallel (i.e., in the same print) with the same mAb (panel C). For both the classical sandwich and premix assay formats on rhPGRN, the assignment of analyte/ligand mAb pairs as blockers or non-blockers was generally clear-cut, based on the lack of response or an obvious response, respectively.

**Figure 4:**
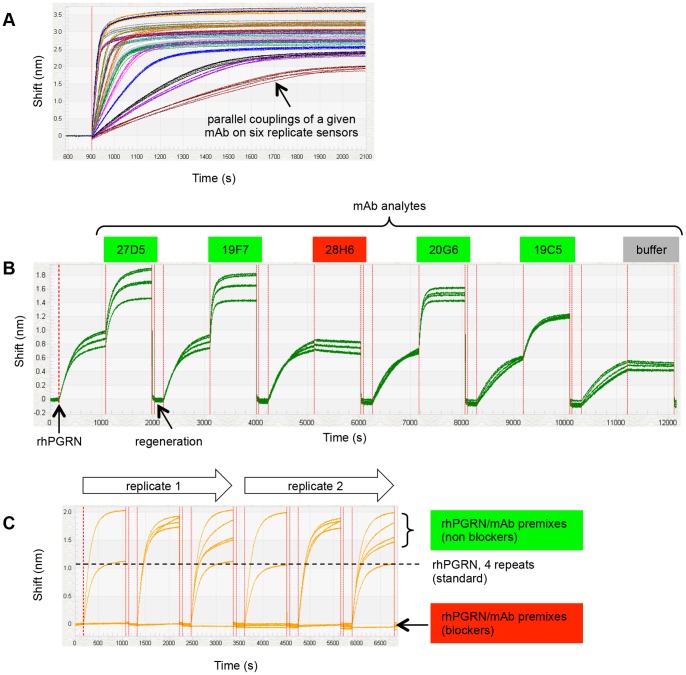
BLI analysis of sixteen anti-hPGRN mAbs using complementary epitope binning assay formats. (A) Overlay plot for the coupling of 96 mAbs on parallel sensors (six replicate sets of sixteen mAbs). (B) Sensorgrams obtained from a classical sandwich assay on six sensors coupled with mAb 1B8 (the sensorgrams cluster into three pairs of curves because the couplings were performed on duplicate sensors in three consecutive runs). Analytes highlighted in green have different epitopes than the ligand (i.e., 1B8 does not block 27D5, 19F7, 20G6, or 19C5) whereas the analyte highlighted in red has an epitope that overlaps with that of the ligand (i.e., 1B8 blocks 28H6). (C) Sensorgrams obtained from a premix assay on six sensors coupled with mAb 2B2 (the same analyte wells were tested in duplicate cycles). Panels B and C are representative of the much larger data sets generated in those experiments. Response data are aligned to zero at the start of the sensorgrams.

We performed a similar epitope binning analysis on rhPGRN using the BLI platform in 96-channel mode. **Figure 4A** shows an overlay plot of 96 sensorgrams that depicts the parallel coupling of sixteen anti-hPGRN mAbs onto six sensors each. We found that parallel couplings of a given mAb onto multiple sensors yielded uniform ligand capacities whereas consecutive couplings of a given mAb showed more variability, likely reflecting cycle-to-cycle differences in the potency of the activation reagents used and/or the gradual loss of a mAb's activity upon prolonged exposure to the low ionic strength coupling buffer. Regardless, the binning result obtained in a classical sandwich assay format on a given mAb ligand was independent of its capacity (**Figure 4B**), provided the binding response was clearly above instrument noise (i.e., even low capacity surfaces yielded useful binning data). In a classical sandwich assay format, a maximum of five different mAb analytes could be run unattended, as limited by the autosampler's capacity (**Figure 1B**). Since we had sixteen mAb analytes, we analyzed fifteen of them in a total of three separate experiments on the same sensors. To extend the scope of the classical sandwich assay on the BLI system to a 96×96 analyte-on-ligand interaction matrix would require 24 separate experiments if one microplate is used to accommodate four mAb analytes (since each mAb analyte must be dispensed into 96 replicate wells) and the second microplate is dedicated to common reagents. Not only would it be cumbersome to prepare 24 plates of mAb analytes and manually exchange these plates, one by one, until all 96 mAb analytes had been addressed, evaporation would limit the number of times the reagent plate could be re-used (see **Table 1**).

The BLI's unique dip-and-read format enables the parallel analysis of a diverse set of interactions because analyte/ligand pairs are entirely independent of one another. To demonstrate this versatility, which is a unique and significant advantage of BLI, we performed a premix assay on the same panel of sixteen anti-hPGRN mAbs in an hour by coupling each mAb onto six sensors and dipping all 96 mAb-coated sensors into samples of rhPGRN premixed with buffer or a mAb, where the binding responses of the rhPGRN+buffer samples served as the “standard” against which the responses of the rhPGRN+mAb premixes were compared on a per ligand basis. Three binding cycles in 96-channel mode therefore addressed all 256 pairwise permutations required to test each mAb as both analyte and ligand along with duplicates of the standard. For this type of experiment, it was important that a uniform ligand capacity was achieved on replicate sensors that were coupled with a given mAb, so we performed all couplings in parallel. Assigning analyte/ligand mAb pairs as blockers or non-blockers was straightforward due to the high quality of the BLI data, as shown in **Figure 4C**.

**Figure 5:**
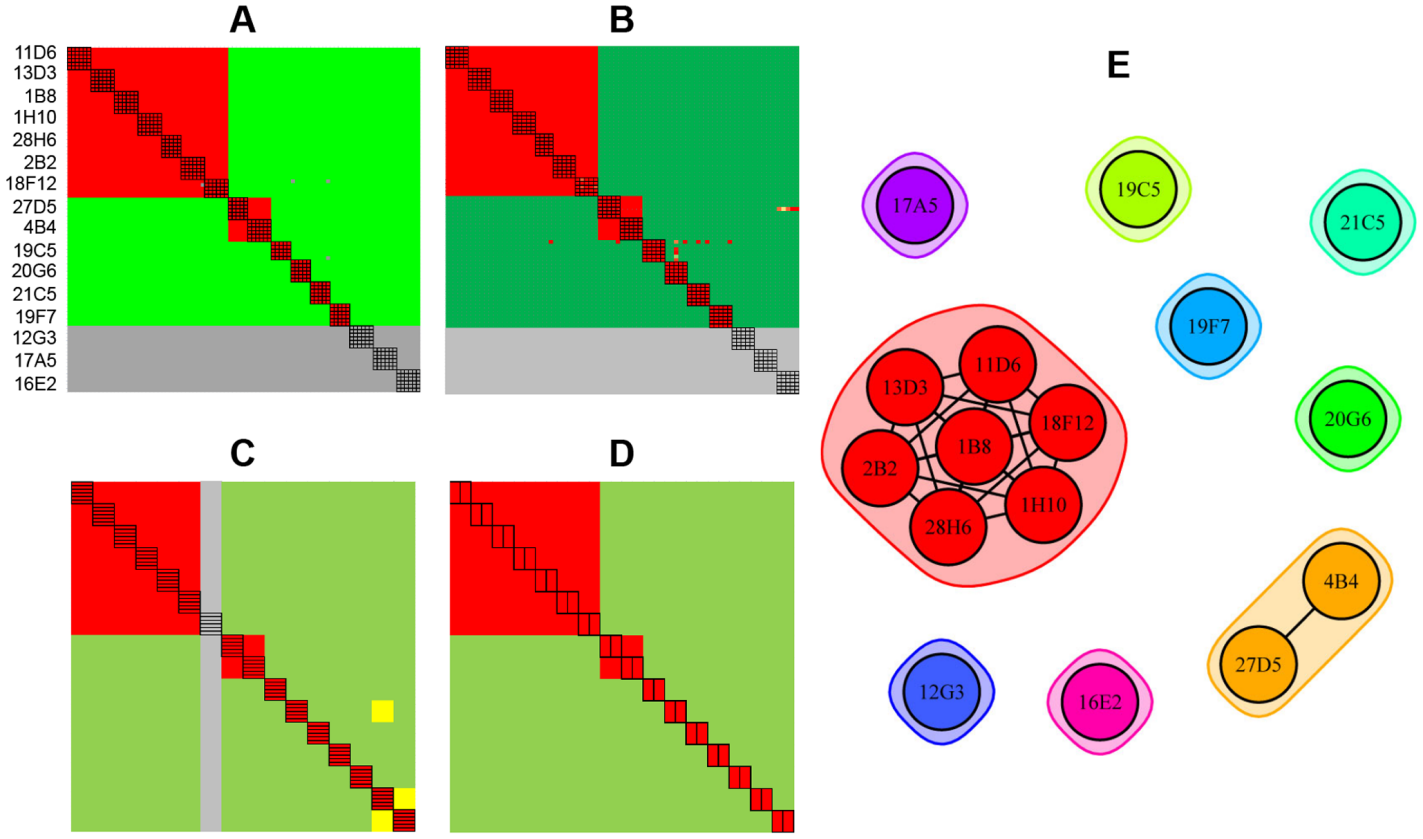
Technology comparison of epitope binning assays on sixteen anti-hPGRN mAbs using complementary assay formats. Heat maps for (A) SPRi – classical sandwich, (B) SPRi – premix, (C) BLI – classical sandwich, and (D) BLI – premix experiments. (E) Node plot of the deduced bin assignments from panels A–D. All experiments were conducted on a 96-ligand array (i.e., sixteen mAbs coupled onto six surfaces each). Sixteen mAbs were each used as analyte (A) up to six times, (B) up to five times, (C) once, and (D) twice. In the heat maps, the rows represent the ligands and the columns represent the analytes, in the same order. Analyte/ligand pairs are assigned as blocked (red), not blocked (green), or ambiguous (yellow), and self-blocks are outlined with a black box. Panels A, B and D each represent the results for a single experiment, whereas panel C represents the consolidated results from three separate experiments on the same sensors, because the autosampler capacity allowed for a maximum of five mAb analytes per experiment. Grey rows in panels A and B indicate inactive ligands and the grey columns indicate mAbs that were not tested as analyte.

**Figure 5** summarizes the epitope binning results obtained for the four experiments described above on rhPGRN. All experiments were conducted on a 96-ligand array, comprising sixteen mAbs each coupled onto six surfaces. In the SPRi experiments (**Figure 5A** – classical sandwich and **Figure 5B** – premix), the blocking status of each mAb pair was tested multiple times because each mAb analyte was injected up to six times, thereby testing every cross-block up to 72 times and every self-block up to 36 times (indicated by the black boxes). Due to BLI's significantly lower unattended throughput and higher analyte consumption (**Table 1**), we analyzed each mAb analyte once (**Figure 5C**) or twice (**Figure 5D**), respectively on a 96-ligand array. **Figure 5E** shows a node plot of the epitope bins determined from both assay formats using both technologies. Node plots can be interpreted by following some simple rules. It is assumed that all mAbs within a node plot have been tested comprehensively for pairwise blocking against one another. Therefore, a chord between two mAbs indicates a blocking relationship and no chord indicates no blocking. Epitope bins are inscribed by the envelopes. As indicated by the nine separate groupings, the sixteen anti-hPGRN mAbs fell into nine non-overlapping bins, i.e., no bin blocked any other bin such that a sandwich pair could be formed across any two bins. Seven mAbs were in unique bins by themselves, while the remaining nine mAbs were divided over two additional bins. Taken together, these results demonstrate that the same binning outcome was obtained regardless of the biosensor platform used or the epitope binning assay format employed.

### High-throughput epitope binning assays yield exquisite resolution of epitope bins

**Figure 6:**
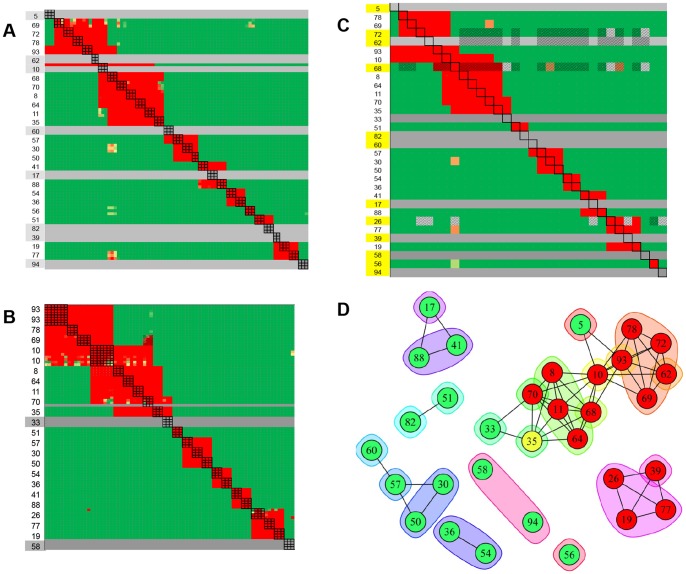
SPRi analysis of anti-IsdB mAbs using a classical sandwich epitope binning assay format. (A and B) Heat maps for two independent experiments on intersecting panels of mAbs. (C) Merged heat map of the 32 unique mAbs condensed to one cell per mAb pair. Grey rows represent inactive ligands and mAbs highlighted in yellow were not represented in both experiments. (D) Node plot of the epitope bin assignments deduced from panel C (mAbs are colored by their functional activity, where green - not blocked, red - block and yellow - partial blocker; see Figure 12). MAbs 5, 17, 33, 39, 58, 60, 62, 82, and 94 were inactive as ligand (i.e., orphan analytes), so were not tested for cross-blocking against one another. Therefore, every orphan is in its own bin (i.e., each is inscribed by its own envelope) to indicate that the missing orphan/orphan cross-blocking information could change their bin assignments. For example, mAbs 19, 26, 39 and 77 mutually cross-block one another so tentatively belong to the same bin, but mAb 39 is inscribed by its own envelope to indicate that it is an orphan analyte. Similarly mAbs 17, 41, and 88 mutually cross-block one another, but mAb 17 is inscribed by its own envelope because it is an orphan analyte. Orphans 58 and 94 tentatively belong to the same bin because they were not blocked by any ligand, but there is no chord connecting them because the 58/94 cross-blocking information is missing.

Unlike our study of anti-hPGRN mAbs described above, our epitope binning experiments on a panel of 63 unique anti-IsdB mAbs (analyzed in a series of experiments on smaller, intersecting subsets; see **Tables 2** **and** **3**) revealed a high frequency of bins that overlapped with other bins and therefore provided a model system for demonstrating how high-throughput binning can yield exquisite discrimination of epitopes. **Figure 6A** shows the heat map for 29 mAbs, each analyzed in triplicate as both analyte and ligand in a classical sandwich assay format by SPRi. Seven of these mAbs were inactive as ligand on all three spots tested and mAb 10 was inactive on two of the three printed spots (inactive ligands are represented by the grey rows). **Figure 6B** shows the heat map for a similar experiment conducted on an independent ligand array comprising 22 unique mAbs, which included 19 mAbs that had shown good ligand activity in the previous experiment. Twenty of these mAbs were printed onto four spots each and two (mAbs 10 and 93) were printed onto eight spots each to complete a 96-ligand array. **Figure 6C** shows the merged heat map for the 32 unique mAbs tested across these two experiments and **Figure 6D** shows a node plot that represents the deduced epitope bins and their inter-bin relationships. Orphan analytes (i.e., mAbs that were inactive as ligand; 5, 17, 33, 39, 58, 60, 62, 82, and 94, as shown by the grey rows) introduce gaps into the heat map because they were neither tested for self-blocking nor cross-blocking against one another. While tentative bins can be assigned to the orphan analytes based on their blocking against the active ligands, further experiments would be required to determine whether the missing orphan/orphan cross-blocking information would alter those assignments; thus, each orphan is inscribed by its own envelope. Orphans 58 and 94 were assigned to the same bin because they exhibited the same blocking profile, i.e., they were not blocked by any ligand, but there is no chord between them because their ability to block one another was not determined. Thus, we assigned 32 mAbs to 21 epitope bins. All node plots for anti-IsdB mAbs are color-coded according to each mAb's functional activity, as determined by their ability to block (red), or not block (green), rIsdB's binding to hemoglobin (Hb), which is the natural ligand for native IsdB and will be discussed in more detail later.

**Table 2:** Summary of the epitope binning study on the anti-IsdB mAbs described in Figures 6, 7, 8, 9, 10, 11.

Figure	Platform	Format	No. mAbs in panel	Bins
**6**	SPRi	Classical	32	21
**7**	BLI	Classical	41	19
**8**	SPRi/BLI	Classical	21	14
**9**	BLI	Tandem	43	25
**10**	BLI	Classical/Tandem	27	15
**11A**	BLI	Tandem	16	13
**11B**	SPRi/BLI	Classical/Tandem	6	4

**Table 3:** Numerical list of the 63 unique anti-IsdB mAbs (named #3-95) represented in the node plots shown in Figures 6, 7, 8, 9, 10, 11.

Fig.6	Fig.7	Fig.8	Fig.9	Fig.10	Fig.11A	Fig.11B
	3					
5	5	5	5	5		
					6	
	7		7	7		
8	8	8	8	8		
					9	
10	10	10	10	10	10	10
11			11			
17	17	17	17	17	17	17
	18		18	18		
19	19	19				
			20			
	21		21	21		
					22	
26			26			
30	30	30	30	30		
					31	
			32			
33	33	33	33	33		
	34				34	
35			35			
36	36	36	36	36	36	36
39			39			
41	41	41				
	43					
	44					
	45				45	
			46			
	47					
			48			
50			50			
51			51			
	53		53	53	53	
54	54	54			54	
	55					
56			56			
57	57	57	57	57	57	57
58			58			
	59					
60	60	60	60	60	60	60
62			62			
64	64	64				
	65		65	65		
	66		66	66		
	67		67	67	67	
68						
69	69	69	69	69	69	69
70	70	70	70	70		
			71			
72	72	72				
77	77	77	77	77		
78	78	78	78	78		
	79		79	79		
82	82	82	82	82		
			83			
88	88	88	88	88		
					89	
	90		90	90		
	91		91	91		
	92		92	92		
93	93	93	93	93		
94			94			
	95					

A different subset of mAbs was analyzed per experiment. The six mAbs (#10, 17, 36, 57 60, and 69) common to all experiments, are represented in Figure 11B.

**Figure 7:**
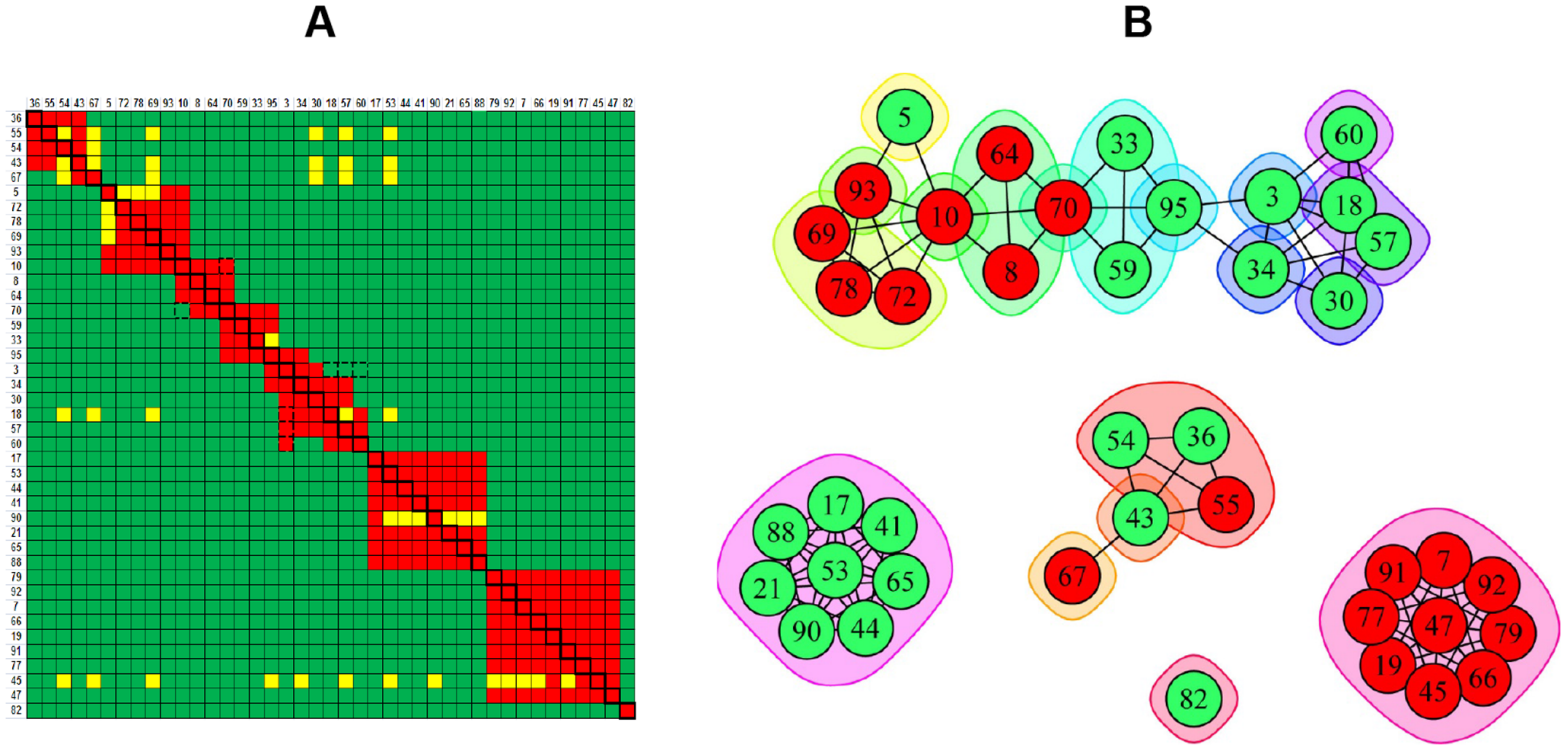
BLI analysis of 41 anti-IsdB mAbs using a classical sandwich epitope binning assay format. (A) Heat map and (B) node plot.

We extended the analysis of our anti-IsdB mAbs to a larger panel comprising 41 mAbs (some of which were also represented in **Figure 6**) and performed a classical sandwich assay on them by BLI. To minimize ligand attrition and thus increase the confidence of our bin assignments, we tailored the regeneration solution to the needs of individual coupled mAbs, which is a unique and significant advantage of the BLI approach because sensors are not linked to one another and so can dip into discrete sample wells. **Figure 7** shows the results of this analysis represented as both a heat map (panel A) and a node plot (panel B). Examining the heat map shows that some mAb pairs gave an ambiguous result in both directions (e.g., 54/67, 5/69, 5/72, and 5/78, indicated by the yellow cells), while other pairs gave a conflicting result dependent upon the order of addition (e.g., 10/70, 3/18, 3/57, and 3/60, indicated by the cells with a dotted border). Both scenarios introduce uncertainty into the bin assignments and warrant further experiments to clarify the result. In the case of a conflicting blocking result between two mAbs, we connect them with a chord in the node plot because a blocking relationship is observed, even if it is only in one direction of the heat map. In this example, we assigned 41 mAbs to 19 epitope bins. Of these bins, only three did not block any other bin, while the remaining mAbs fell into two clusters, one of which represented an intricate network of bins.

**Figure 8:**
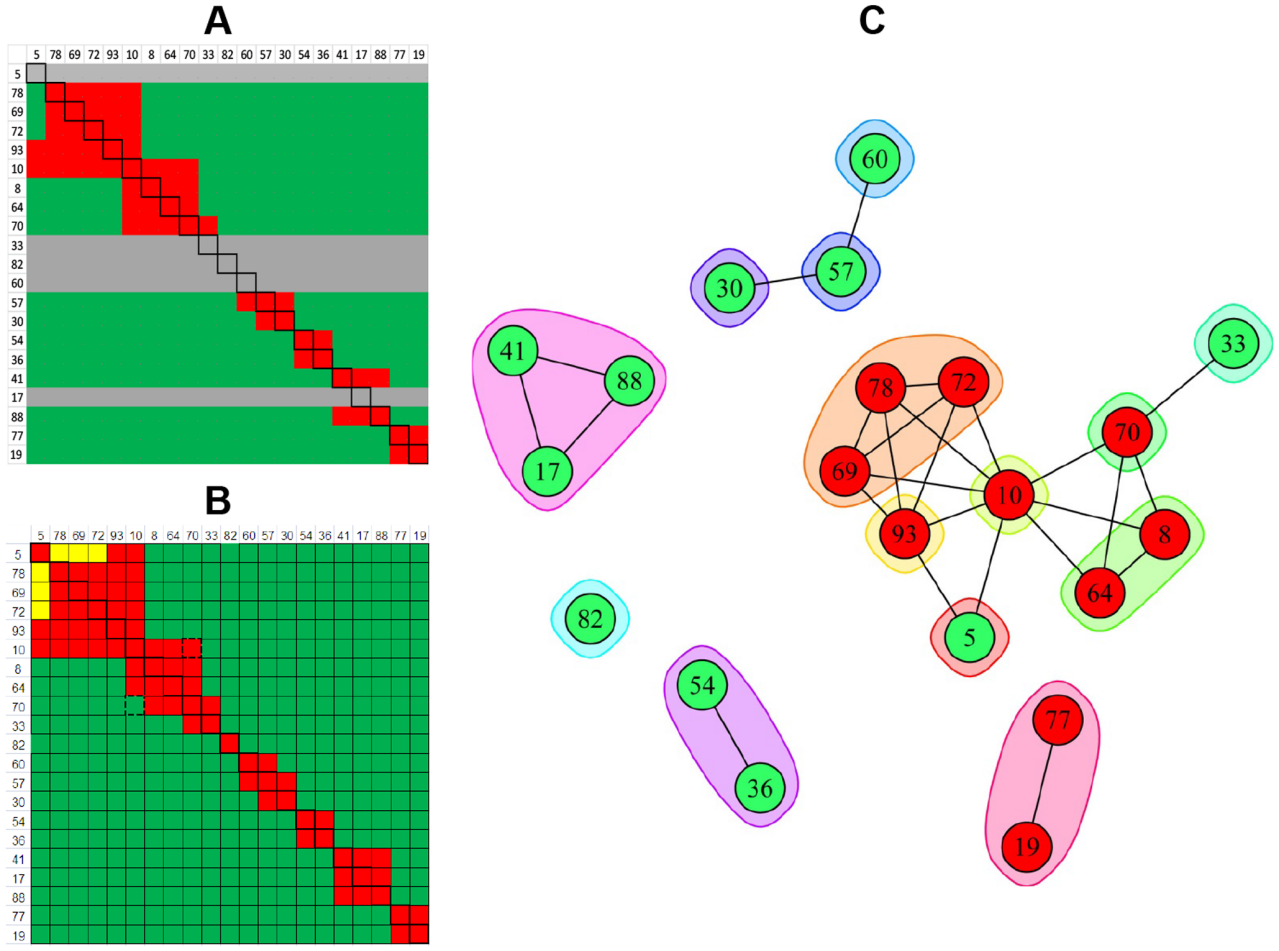
Technology comparison of classical sandwich assays performed on 21 anti-IsdB mAbs. Heat maps obtained from (A) SPRi (see Figure 6C) and (B) BLI (see Figure 7A). (C) Node plot.

To facilitate a direct comparison of the results obtained for classical sandwich assays using SPRi and BLI technologies, the heat maps for 21 mAbs that were represented in both experiments are shown in **Figure 8A** and **Figure 8B**, respectively. It is noteworthy that five ligands (5, 17, 33, 60, and 82) were inactive by SPRi (indicated by the grey rows in **Figure 8A**) but active by BLI. Their activity was preserved in the BLI assay because a gentler regeneration condition was employed relative to that used for most of the other ligands, i.e., 15 mM phosphoric acid instead of 75 mM phosphoric acid. Thus, the open configuration of the BLI platform enabled us to minimize ligand attrition by tailoring the regeneration condition per mAb-coated sensor, whereas the SPRi's single flow cell approach relied on the use of a universal regeneration condition for a given ligand array which damaged some ligands. **Figure 8C** shows the node plot that was deduced by combining the information from both technologies.

### The BLI platform allows for ad hoc sensor replacement and on-line reloading of an array, which makes the in tandem epitope binning assay format feasible

**Figure 9:**
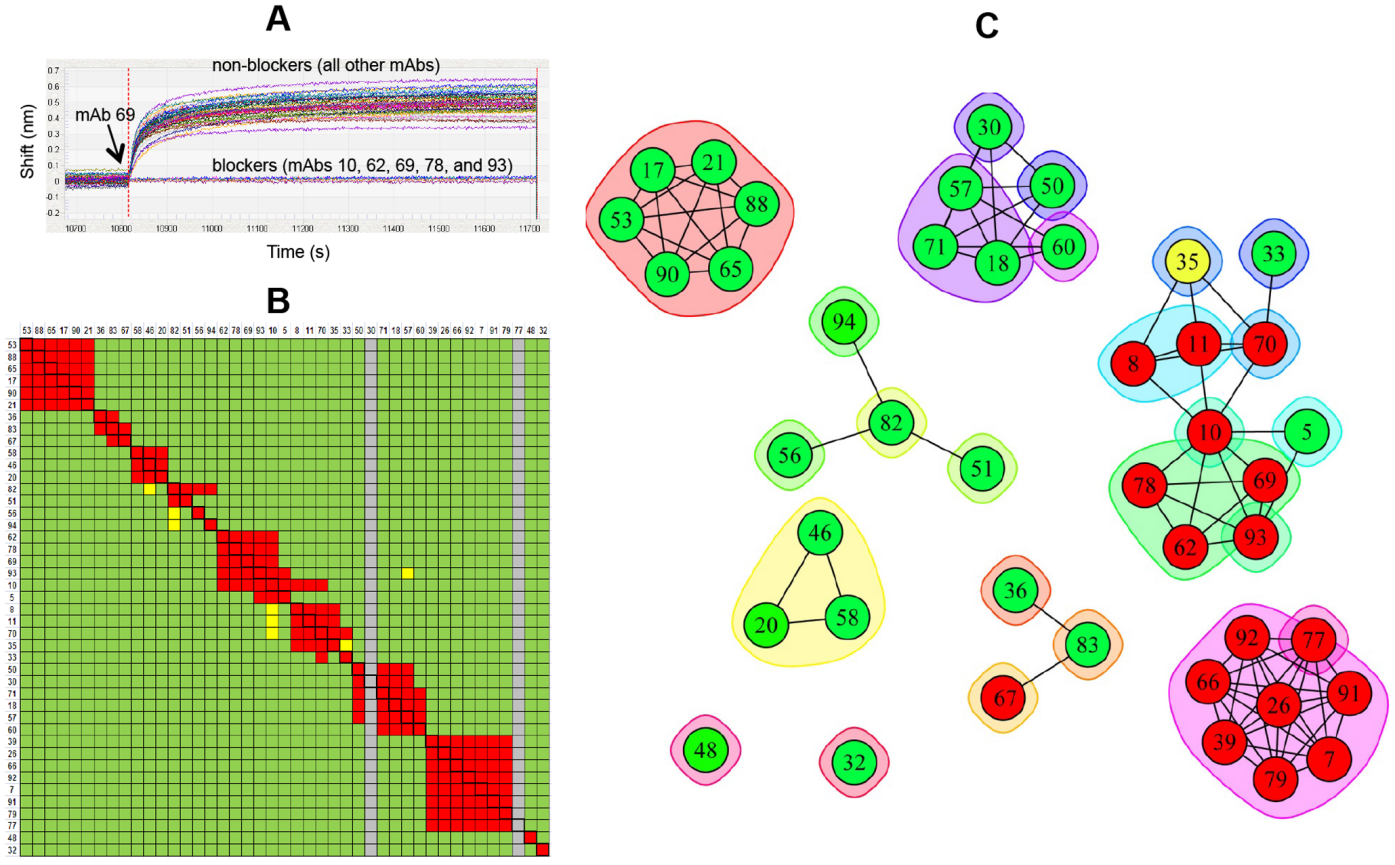
BLI analysis of 43 anti-IsdB mAbs using an in tandem epitope binning assay format. (A) Response data for mAb 69 binding as analyte to anti-His-captured rIsdB that was saturated by a 48-mAb array, (B) heat map, and (C) node plot.

To complement our classical sandwich data and bypass ligand attrition issues, we performed an in tandem epitope binning assay on 43 unique anti-IsdB mAbs using BLI. By employing a reversible capture of the antigen via a pre-immobilized anti-His mAb, neither the antigen nor the mAbs were regenerated, which preserved their native activity. **Figure 9A** shows an overlay plot of the sensorgrams obtained for mAb 69 binding as analyte to the anti-His captured antigen that was first saturated by an array of 48 mAbs (representing 43 unique clones); in this case, mAb 69 is clearly blocked only by mAbs 10, 62, 69, 78, and 93. **Figure 9B** shows the heat map for a comprehensive pairwise analysis of 43 mAbs and **Figure 9C** shows a node plot of the bin assignments. Neither mAb 30 nor mAb 77 were tested as analyte due to their low sample availability, so are “orphan ligands” in this assay (represented by the grey columns). While the 30/77 cross-block was not tested, there is sufficient information from the heat map to assign mAb 30 and mAb 77 to different bins. The BLI's autosampler limited an unattended run to only eight mAb analytes (since each mAb analyte was distributed into 48 wells of the sample plate), so six separate experiments were needed to address a full 48×48 interaction matrix when the instrument was used in 96-channel mode.

**Figure 10:**
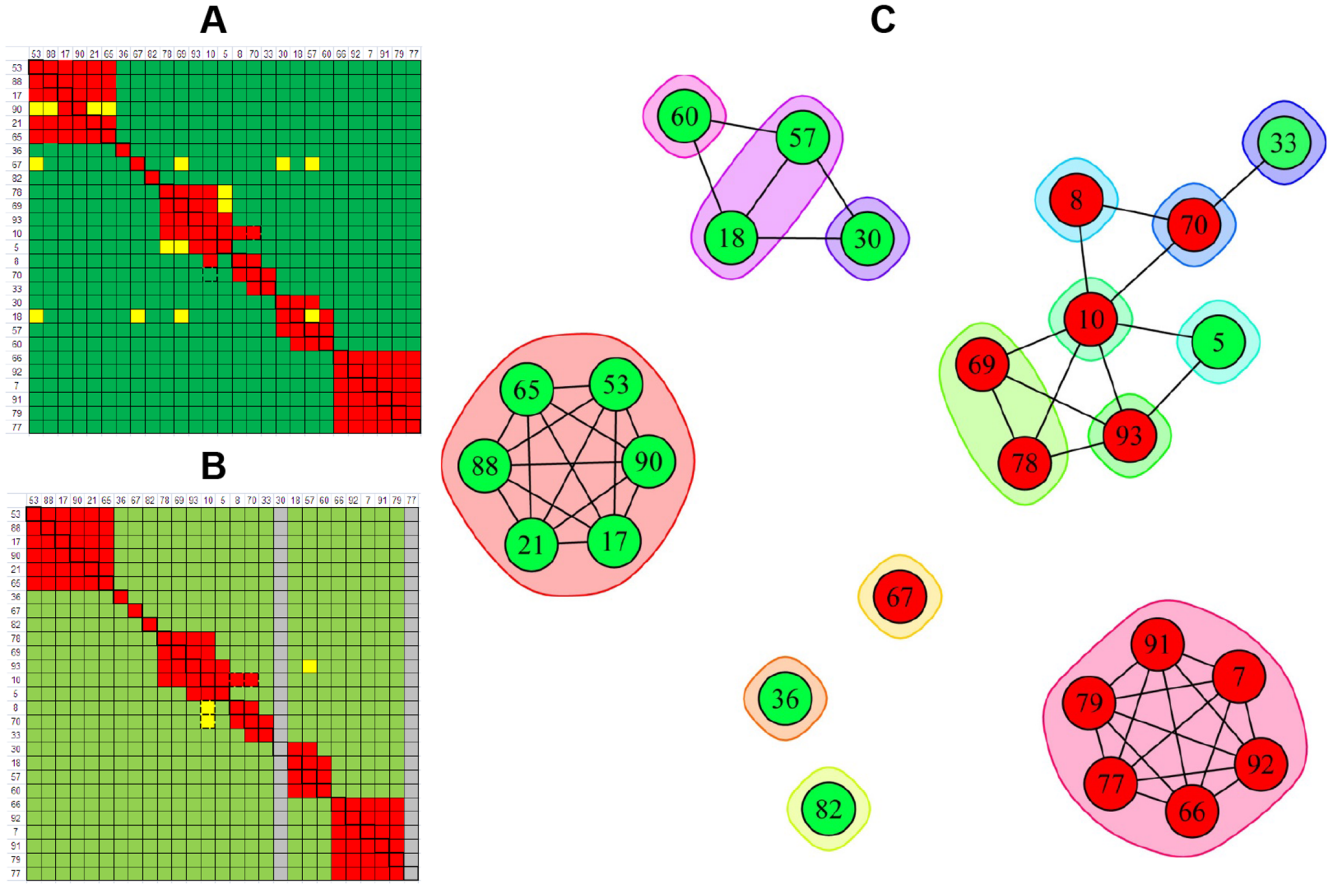
BLI analysis of 27 anti-IsdB mAbs using complementary epitope binning assay formats. Heat maps for (A) classical sandwich and (B) in tandem approaches. (C) Node plot.

**Figure 11:**
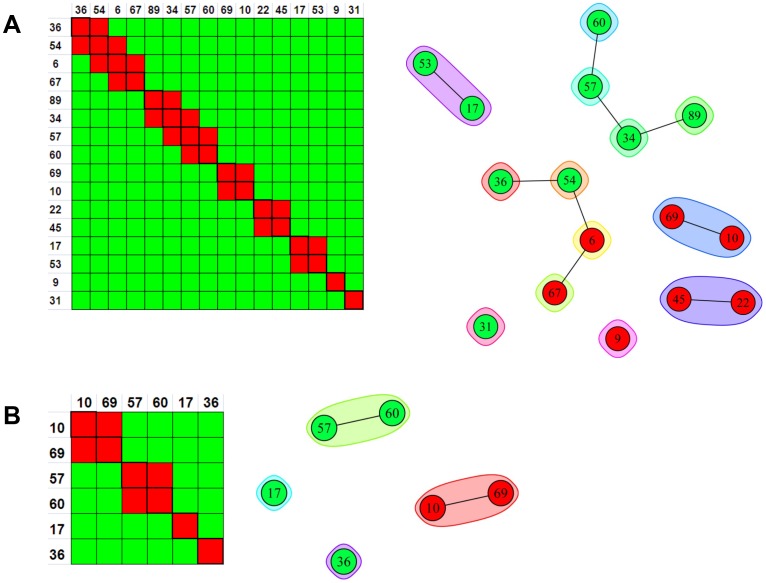
Heat maps and node plots for epitope binning experiments on small panels of anti-IsdB mAbs. (A) BLI analysis of sixteen mAbs using an in tandem epitope binning assay format; the same result was obtained from two independent experiments in which the antigen was captured via anti-His or anti-Flag surfaces. (B) Binning result for the six mAbs that were common to all rIsdB experiments shown in Figures 6–11A.

A direct comparison of the heat maps obtained for 27 anti-IsdB mAbs when studied by BLI using a classical sandwich assay format (**Figure 10A**) or an in tandem assay format (**Figure 10B**) shows that the same bin assignments were deduced, irrespective of the assay format used, as visualized in the node plot shown in **Figure 10C**. We also performed an in tandem style epitope binning experiment on sixteen carefully selected anti-IsdB mAbs to demonstrate that a small panel of mAbs can yield overlapping bins if it contains sufficient epitopic diversity (**Figure 11A**). The same heat map and node plot describing thirteen epitope bins were deduced, regardless of whether we employed an anti-His or anti-Flag capture of the antigen (data not shown), confirming that neither of the two immobilization methods perturbed the epitopes within our mAb panel. To demonstrate that we obtained consistent binning results across all our rIsdB experiments, **Figure 11B** shows that the same heat map and node plot are recovered from **Figures 6** **-** **11A** if one extracts only the data for the six mAbs that were common to all those experiments.

### Epitope bins correlate with functional activity

**Figure 12:**
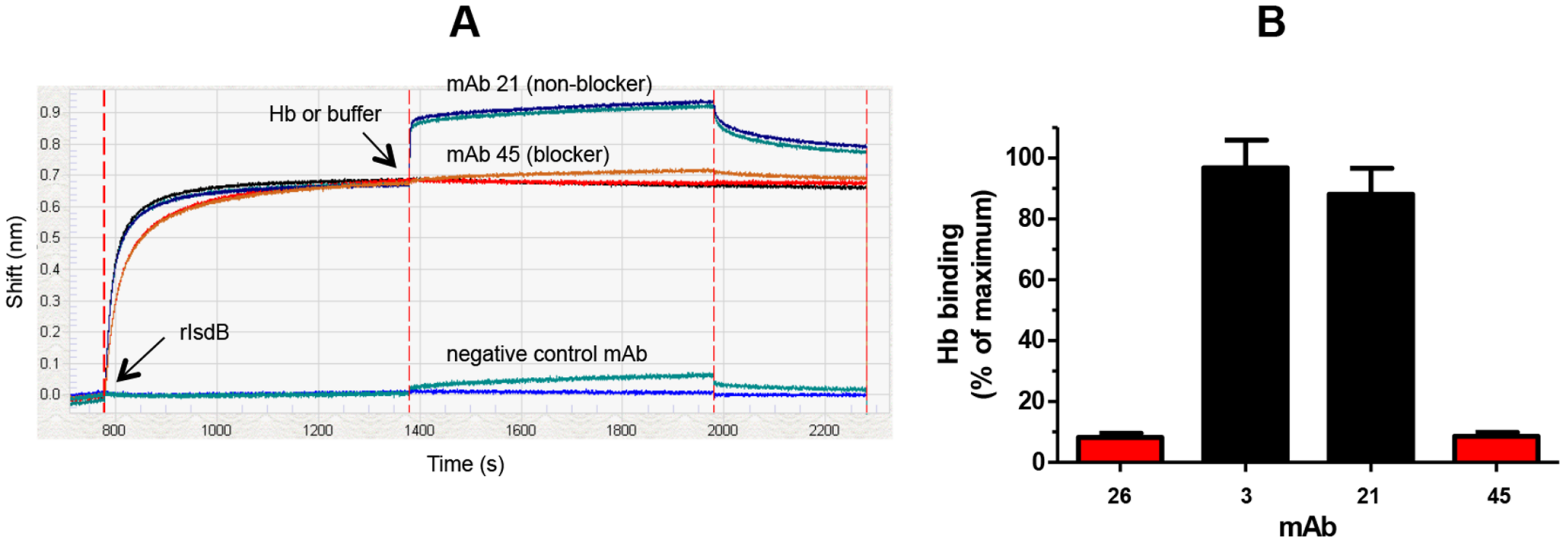
Correlation of epitope bin with functional activity. Example of Hb-blocking data obtained for select anti-IsdB mAbs using (A) rIsdB in a BLI assay and (B) native IsdB in a cell-based assay (the bars and error bars represent the mean and standard deviation for three independent measurements).

As stated earlier, Hb is the natural ligand for IsdB [22]. Using the BLI platform, we screened 63 unique anti-IsdB mAbs to determine whether they blocked Hb binding (**Figure 12A**) and then chose a smaller subset on which we performed a functional cell-based blocking assay (**Figure 12B**). We observed a strong correlation between our Hb-blocking data obtained on recombinant and native IsdB based on fifteen of the sixteen mAbs tested in the cell-based assay producing the same result as the biosensor. The outlier, mAb77, was a clear blocker when tested multiple independent times in the biosensor assay, but was assigned as a non-blocker after a single test in the cell-based assay because it gave only 20% reduction in Hb binding. All the node plots in our rIsdB study (see **Figures 6** **,** **7** **,** **8** **,** **9** **,** **10** **,** **11**) are color-coded according to each mAb's ability to block the rIsdB/Hb interaction, as determined by BLI. Our results reveal an excellent correlation between epitope bin and Hb-blocking activity since all mAbs in a given epitope bin or network of inter-connected bins exhibited the same blocking profile towards Hb. Moreover, several non-overlapping epitope bins showed functional activity, suggesting that mAbs with different epitopes may block Hb via different mechanisms of action.

## Discussion

### The main advantages of the SPRi platform are its exceptionally low sample requirement, facile sample preparation, and unparalleled unattended throughput

In evaluating two 96-ligand array-based technologies, we found that they were complementary to one another. One of the main advantages of using single flow cell microarray-based SPRi is its exceptionally low sample consumption, which is not only appealing for conserving precious samples, but simplifies the sample preparation (**Table 1**). Since similar mAb concentrations were used on both platforms, sample consumption can be compared in terms of volume. While both platforms require only 100 μl per mAb to array 96 ligands, the SPRi platform consumes almost 100-fold less mAb analyte than the BLI platform to perform a comprehensive epitope binning experiment on 96 mAbs. Thus, the SPRi assay consumes a single 120 μl per mAb analyte whereas BLI requires 10 ml per mAb analyte because it must be distributed as ninety-six 100 μl-aliquots into a 384-well sample plate. Another significant difference between the SPRi and BLI platforms is their unattended throughput, as dictated by their respective autosamplers. The SPRi platform has unparalleled unattended throughput because 96 analytes can be injected over a 96-ligand array, thereby addressing 9,216 analyte/ligand interactions per experiment, with a runtime of approximately 30 hours (**Table 1**). In contrast, the number of analytes that can be tested by BLI against a 96-ligand array in the context of an epitope binning experiment is limited to five or less, depending upon the assay format used, since all samples (including analytes and common reagents such as antigen, buffer, and regeneration solutions) are accommodated within two 384-well microplates. A higher unattended throughput can be achieved by integrating the BLI system to a robot that automates multi-plate analyses, but this comes at an additional cost to the user. However, even with a robotic system, the unattended runtime of a BLI assay is still limited because of sample evaporation issues.

### The BLI platform is highly versatile and enables the parallel analysis of 96 independent analyte/ligand pairs, ad hoc sensor replacement, and on-line reloading of an analyte- or ligand-array

The open configuration of the BLI platform makes it amenable to the analysis of diverse interactions and various assay formats. Processing a collection of discrete sensors in parallel allows for the simultaneous analysis of 96 entirely independent analyte/ligand interactions, which can be exploited to perform rapid epitope binning experiments on small panels of mAbs. For example, we demonstrated that a 16×16 interaction matrix can be addressed using a premix assay format in an hour using just three 20-minute binding cycles in 96-channel mode (**Figure 4C**), which could have been reduced to 30 minutes or less by using a shorter binding cycle. Indeed, the largest panel that can be studied in a single BLI experiment when used in 96-channel mode (without robotic integration) is 23 mAbs by using a premix format because it requires only three steps per binding cycle (i.e., buffer baseline, analyte binding, and regeneration). To address an entire 23×23 interaction matrix, each mAb would be coupled onto four sensors and the premixed samples, buffer, and regeneration would be distributed in an appropriate manner across the two sample plates. While this would require a complicated sample layout, the runtime would be only an hour using six ten-minute binding cycles. In contrast, performing a similar analysis using SPRi would take several hours using standard injection times. Another appealing feature of BLI is its ability to load an array on-line, which makes BLI amenable to the in tandem binning assay format. BLI also enables the *ad hoc* replacement of sensors and various tip types can be processed in parallel to diversify the assay further.

### The number of epitope bins that can be resolved in a given experiment depends upon the epitopic diversity within the panel of mAbs being studied

**Table 2** shows that different numbers of epitope bins were deduced from our rIsdB experiments according to the epitopic diversity represented within each panel of mAbs tested. The binning assignments were concordant across these experiments, even though the resolution of the binning result varied. For example, if one extracts from **Figures 6, 7, 8, 9, 10, 11A** only the data for the subset of mAbs that was common to all rIsdB experiments (mAbs 10, 17, 36, 57, 60, and 69; see **Table 3**), four epitope bins are deduced, as shown by the simple node plot in **Figure 11B**. While mAbs 10 and 69 appear to belong to the same bin when analyzed within a panel of six mAbs, their ability to form different blocking relationships with other mAbs is revealed when they are analyzed within the context of a larger panel of mAbs (see **Figures 6** **,** **7** **,** **8** **,** **9** **,** **10**). MAb 10 has a broadly blocking epitope because it is uniquely positioned within the center of an intricate network of blocking interactions, whereas mAb 69's epitope is narrower. Therefore, the larger the panel of mAbs that can be analyzed in a given experiment, the higher the discriminating power of the assay in terms of resolving the epitopes of two mAbs that cross-block one another, because cross-blocking is *necessary but not sufficient* for two mAbs to belong to the same epitope bin.

From a theoretical perspective, a given antigen presents an infinite number of epitopes and the number of unique mAbs that can bind a given epitope is also potentially infinite. In practice, the maximum number of bins that can be determined in a given epitope binning experiment is equal to the number of mAbs in the test panel. The actual number of bins determined experimentally is a relative number that depends on the epitopic diversity of mAbs represented within the test set and typically yields fewer bins than the theoretical maximum when working with large panels of mAbs. In general, only a small number of the total discovered epitope bins in a given project will be functionally relevant. Therefore, one of the most significant benefits to this approach is rapid triaging of a large panel of mAbs early in the discovery process in order to narrow down the number of candidates that are studied further in other assays. This allows mAbs within a functional bin to be prioritized based on other important characteristics like biophysical properties, high specificity for the target and species cross-reactivity for the same target (e.g., mouse, cynomolgus monkey, and human). Our study has demonstrated that label-free epitope binning assays that are both high-throughput and high-resolution can guide our discovery and development of therapeutic mAbs.

## References

[1] Klein C, Lammens A, Schafer W, Georges G, Schwaiger M, et al (2013) Epitope interactions of monoclonal antibodies targeting CD20 and their relationship to functional properties. MAbs 5: 22–33. 23211638 10.4161/mabs.22771 PMC3564883

[2] Markovitz RC, Healey JF, Parker ET, Meeks SL, Lollar P (2013) The diversity of the immune response to the A2 domain of human factor VIII. Blood 121: 2785–2795. 23349389 10.1182/blood-2012-09-456582 PMC3617638

[3] EL-Manzalawy Y, Honavar V (2010) Recent advances in B-cell epitope prediction methods. Immunome Research (Suppl 2):S2. 10.1186/1745-7580-6-S2-S2 PMC2981878 21067544

[4] Emde A, Pradeep C-R, Ferraro DA, Ben-Chetrit N, Sela M, et al (2011) Combining epitope-distinct antibodies to HER2: cooperative inhibitory effects on invasive growth. Oncogene 30: 1631–1642. 21132012 10.1038/onc.2010.547 PMC3632784

[5] Koefoed K, Steinaa L, Søderberg JN, Kjær I, Jacobsen HJ, et al (2011) Rational identification of an optimal antibody mixture for targeting the epidermal growth factor receptor. MAbs 3: 584–595. 22123060 10.4161/mabs.3.6.17955 PMC3242845

[6] Jamnani FR, Rahbarizadeh F, Shokrgozar MA, Ahmadvand D, Mahboudi F, et al (2012) Targeting high affinity and epitope-distinct oligoclonal nanobodies to HER2 over-expressing tumor cells. Experimental Cell Research 318: 1112–1124. 22440788 10.1016/j.yexcr.2012.03.004

[7] Robak T, Windyga J, Trelinski J, von Depka Prondzinski M, Giagounidis A, et al (2012) Rozrolimupab, a mixture of 25 recombinant human monoclonal RhD antibodies, in the treatment of primary immune thrombocytopenia. Blood 120: 3670–3676. 22915649 10.1182/blood-2012-06-438804

[8] Spangler JB, Manzari MT, Rosalia EK, Chen TK, Wittrup KD (2012) Triepitopic Antibody fusions inhibit Cetuximab-resistant BRAF and KRAS mutant tumors via EGFR signal repression. J Mol Biol 422: 532–544. 22706026 10.1016/j.jmb.2012.06.014 PMC4041985

[9] Rich RL, Myszka DG (2007) Higher-throughput, label-free, real-time molecular interaction analysis. Anal Biochem 361: 1–6. 17145039 10.1016/j.ab.2006.10.040

[10] Eddings MA, Miles AR, Eckman JW, Kim J, Rich RL, et al (2008) Improved continuous-flow print head for microarray deposition. Anal Biochem 382: 55–59. 18703010 10.1016/j.ab.2008.07.031

[11] Christensen LH, Holm J, Lund G, Riise E, Lund K (2008) Several distinct properties of the IgE repertoire determine effector cell degranulation in response to allergen challenge. J Allergy Clin Immunol 122: 298–304. 18572230 10.1016/j.jaci.2008.05.026

[12] Song HY, Zhou X, Hobley J, Su X (2012) Comparative study of random and oriented antibody immobilization as measured by dual polarization interferometry and surface plasmon resonance spectroscopy. Langmuir 28: 997–1004. 22126088 10.1021/la202734f

[13] Aldaz-Carroll L, Whitbeck JC, Ponce de Leon M, Lou H, Pannell LK, et al (2005) Physical and immunological characterization of a recombinant secreted form of the membrane protein encoded by the vaccinia virus L1R gene. Virology 341: 59–71. 16083934 10.1016/j.virol.2005.07.006

[14] Krummenacher C, Baribaud I, Ponce de Leon M, Whitbeck JC, Lou H, et al (2000) Localization of a binding site for herpes simplex virus glycoprotein D on herpesvirus entry mediator C by using antireceptor monoclonal antibodies. J Virol 74: 10863–10872. 11069980 10.1128/jvi.74.23.10863-10872.2000 PMC113165

[15] Gesellchen F, Zimmermann B, Herberg FW (2005) Direct optical detection of protein-ligand interactions. Methods Mol Biol 305: 17–46. 15939992 10.1385/1-59259-912-5:017

[16] Abdiche YN, Lindquist KC, Stone DM, Rajpal A, Pons J (2012) Label-free epitope binning assays of monoclonal antibodies enable the identification of antigen heterogeneity. J Immunol Methods 382: 101–116. 22609372 10.1016/j.jim.2012.05.010

[17] Abdiche YN, Malashock DS, Pinkerton A, Pons J (2009) Exploring blocking assays using Octet, ProteOn, and Biacore biosensors. Anal Biochem 386: 172–180. 19111520 10.1016/j.ab.2008.11.038

[18] Abdiche YN, Lindquist KC, Pinkerton A, Pons J, Rajpal A (2011) Expanding the ProteOn XPR36 biosensor into a 36-ligand array expedites protein interaction analysis. Anal Biochem 411: 139–151. 21168382 10.1016/j.ab.2010.12.020

[19] Sun G, Palmer AF (2008) Preparation of ultrapure bovine and human hemoglobin by anion exchange chromatography. J Chromat B 867: 1–7. 10.1016/j.jchromb.2008.02.014 PMC2430017 18359279

[20] Pishchany G, McCoy AL, Torres VJ, Krause JC, Crowe JE Jr, et al (2010) Specificity for human hemoglobin enhances Staphylococcus aureus infection. Cell Host Microbe 8: 544–550. 21147468 10.1016/j.chom.2010.11.002 PMC3032424

[21] McDevitt D, Francois P, Vaudaux P, Foster TJ (1995) Identification of the ligand-binding domain of the surface-located fibrinogen receptor (clumping factor) of Staphylococcus aureus. Mol Microbiol 16: 895–907. 7476187 10.1111/j.1365-2958.1995.tb02316.x

[22] Torres VJ, Pishchany G, Humayun M, Schneewind O, Skaar EP (2006) Staphylococcus aureus IsdB is a hemoglobin receptor required for heme iron utilization. J Bacteriol 188: 8421–8429. 17041042 10.1128/JB.01335-06 PMC1698231

